# The influence of paternal overweight on sperm chromatin integrity, fertilization rate and pregnancy outcome among males attending fertility clinic for IVF/ICSI treatment

**DOI:** 10.1186/s12884-022-04953-z

**Published:** 2022-08-05

**Authors:** Riffat Bibi, Sarwat Jahan, Tayyaba Afsar, Ali Almajwal, Mohammad Eid Hammadeh, Nawaf W. Alruwaili, Suhail Razak, Houda Amor

**Affiliations:** 1 Department of Animal Sciences, Faculty of Biological Sciences, Quaid-I-Azam University Islamabad, Islamabad, 45320 Pakistan; 2 Department of Community Health Sciences, College of Applied Medical Sciences, King Saud University, Riyadh, Saudi Arabia; 3 Department of Obstetrics, Gynecology and Reproductive Medicine, Saarland University Clinic, Homburg, Germany

**Keywords:** Overweight paternal BMI, Sperm chromatin integrity, Assisted reproductive procedures, Sperm deoxyribose nucleic acid fragmentation index

## Abstract

**Background:**

Low and middle-income countries are facing a rapid increase in obesity and overweight burden, particularly in urban settings. Being overweight in men is associated with infertility and a higher risk to have a low sperm count or no sperm in their ejaculate. Despite potential limitations, this is one of few studies conducted to determine the potential risk of paternal overweight on sperm standard parameters, sperm chromatin integrity and assisted conception outcome including fertilization, embryo quality, cleavage rate, reduce blastocyst development, implantation, and cumulative live birth rate (CLBR).

**Methods:**

A cross-sectional study of 750 infertile couples undergoing assisted reproduction technique at a single reproductive medicine center of Salma Kafeel Medical Centre Islamabad. Sperm from men undergoing ART were analyzed for chromatin integrity using sperm chromatin dispersion assay (SCD), Chromomycin A3 staining (CMA3), and toluidine blue (TB) staining, while other semen parameters were assessed on same day includes; standard semen parameters, reactive oxygen species (ROS), sperm deformity index (SDI), teratozoospermic index (TZI), and hypo-osmatic swelling test (HOST). Paternal body mass index (BMI) < 24.5–20 kg/m^2^ served as the reference group, while the male patients with BMI > 24.5-30 kg/m^2^ were considered to be overweight.

**Results:**

In the analysis of the percentage of spermatozoa with chromatin maturity (CMA3) and chromatin integrity (TB) was reduced significantly in overweight men (*p* < 0.01) compared with a reference group. Increase in paternal BMI correlate with the increase in sperm chromatin damage (SCD *r* = 0.282, TB *r* = 0.144, *p* < 0.05), immaturity (CMA3, *r* = 0.79, *p* < 0.05) and oxidative stress (ROS) (*r* = 0.282, *p* < 0.001). Peri-fertilization effects were increased in oocytes fertilization in couples with overweight men (FR = 67%) compared with normal-weight men (FR = 74.8%), similarly, after univariant regression paternal weight remain predictor of sperm chromatin maturity, successful fertilization and CLBR. In the embryo, developmental stage number of the embryo in cleavage was higher in normal weight men, while day 3 (D3) embryos, percent good quality embryo D3, and blastocyst formation rate were compared able between the groups. The paternal overweight group had significant (*p* < 0.001) increased neonatal birth weight (2952.14 ± 53.64gm; within normal range) when compared with the reference group (2577.24 ± 30.94gm) following assisted reproductive technology (ART). CLBR was higher (*p* < 0.05) in normal weight men compared to couples with overweight male partners. CLBR per embryo transfer and per 2PN was a statistically significant (*p* < 0.05) difference between the two groups. An inverse association was observed in the linear regression model between paternal BMI with fertilization rate and CLBR.

**Conclusion:**

The present study demonstrated the impact of paternal overweight on male reproductive health, as these patients had a higher percentage of immature sperm (CMA3) with impaired chromatin integrity (SCD, TB) in their semen and had decreased fertilization rate, CLBR following assisted reproductive treatments. The present study supports that paternal overweight should be regarded as one of the predictors for fertilization, CLBR and useful for counseling, to consider body mass index not only in women but also for men, in those couples opting for ART treatment, and warrant a poor reproductive outcome in overweight men.

## Plain Summary

Infertility is a global reproductive health issue faced by 10–15% of couples of reproductive ages. An estimated 3.5–16.7% of couples are affected in developing countries and 6.9–9.3% of couples are affected in developed countries. The purpose of this study was to examine the correlation of paternal BMI on semen parameters (concentration, motility, morphology, and vitality), DNA fragmentation, chromatin maturity, and assessment of possible impact of male BMI, sperm DNA fragmentation, chromatin maturity influence on fertilization, embryo quality, live birth rate, and birth weight. Despite potential limitations, this is one of few studies with extensive information on the potential risk of paternal obesity on sperm epigenetics. Before recommending the ART procedure to an overweight couple. It is advisable to encourage weight loss not only to females but also to male partners as it is sagacious to increase the quality of gametes and ART cycles and birth outcomes.

## Background

Infertility is a global reproductive health issue faced by 10–15% of couples of reproductive ages. An estimated 3.5–16.7% of couples are affected in developing countries and 6.9–9.3% of couples are affected in developed countries [1, 2]. Fertility specialists in third-world countries face major difficulty during the investigation of an infertile couple. Due to the limiting social beliefs that the cause of infertility lies in the female. As a consequence of which male partners hardly present themselves for investigation making it difficult to access the true cause of infertility [3, 4]. Male factor infertility prevails in approximately 25% of all such couples [2, 5]. It has been reported that in male partners opting for semen analysis; over 50% of men presented with abnormal semen parameters. It is estimated that the prevalence of male infertility between the age of 15 to 50 years was up to 6% [2, 3, 6]. In recent years’ obesity becomes a key factor contributing to debility in reproductive health indices in both sexes. Superfluous energy alters the regulatory mechanisms of the reproductive system. Persons with obesity have augmented estrogen levels, due to the amplification of aromatase in the adipose tissue; through a negative response loop, men display indications of hypogonadotropic hypogonadism. These hormonal fluctuations, besides augmented oxidative stress, lipotoxicity, and instabilities in the absorptions of adipokines, directly distress the gonads, peripheral reproductive organs, and the embryo [7]. It is generally well accepted that reproductive function highly correlates with the degree of adiposity, nutrition, or metabolic condition related to food intake in human medicine [8, 9]. Paternal BMI Kg/m^2^ < 16.5 (underweight) and > 30 (obesit**y)** were associated with reduced semen quality [8, 10, 11]. Moreover, known acquired factors that contribute to male infertility include infection, immunological factors, trauma or surgical insult to the male reproductive organs, and exposure to toxic chemicals or other materials [1, 2, 12]. Similarly, a direct association was found between men's BMI kg/m.^2^ and semen quality found even after adjustment for reproductive hormones [13]

Semen analysis is a routine and simple method for assessing male fertility status. However, alone it is not sufficient to predict assisted reproductive outcomes [14, 15]. With the development of new predictive tools to identify male fertility potential. Sperm deoxyribonucleic acid fragmentation index is a commonly used technique involving methods such as Terminal deoxynucleotidyl transferase dUTP nick-end labeling (TUNEL) assay, Comet assay, Sperm chromatin structure assay (SCSA), acridine Orange test, and sperm chromatin dispersion (SCD) assay [14, 16]. Identification of DFI through SCD assays is cheaper yet equally reliable when compared with TUNEL assay which is expensive and utilizes advanced equipment [17–19].

Multiple cross-sectional studies and meta-analyses have found inconsistent results of varying correlations between BMI, semen parameters, male reproductive hormones, Sperm DNA fragmentation, chromatin structure, and ART outcome [15, 20]. A systematic review of the literature demonstrates that sperm DNA damage is associated with lower pregnancy rates and pregnancy loss after assisted conception techniques employing in-vitro fertilization (IVF) and Intracytoplasmic insemination (ICSI) [9, 21–23], particularly ICSI where it circumvents the natural defense barriers and allows for fertilization with DNA damaged sperm. Therefore, increasing concerns regarding the health outcomes for the resulting offspring [2, 23, 24]. Besides, other factors, obesity would be the leading cause of lower pregnancy rate and failure of reproductive outcomes. Therefore, the overall health and normal BMI of parents should be considered and of important concern in attaining reproductive outcomes. We aimed to investigate the correlation of paternal BMI on semen parameters (concentration, motility, morphology, and vitality), DNA fragmentation, and chromatin maturity. Furthermore, we investigated the correlation matrix of the possible impact of paternal high BMI on fertilization, embryo quality, live birth rate, and birth weight.

## Methods

### Study design

Study was conducted at the Reproductive Physiology Laboratory, Department of Zoology, Faculty of Biological Sciences, and Quaid-i-Azam University in Islamabad Pakistan. Samples were collected form electronic medical record system at Fertility and Genetic services, SKMC Islamabad, Pakistan. Informed consent was taken from all the participation in the study.

### Ethical approval

The study was approved by the ethical committee of Quaid I Azam University and SKMC Islamabad Pakistan. The ethical approval to conduct this study was obtained from the Ethics Committee of Salma Kafeel Medical Centre Islamabad Pakistan and from the Bio-Ethic committee of the Department of Zoology, Quaid-i-Azam University, Islamabad and was assigned protocol # BEC-FBS-QAU2016-77.

### Participants

Couples undergoing their first ovarian stimulation (who remained unsuccessful in achieving pregnancy after trying for 12 or more months, with male partner BMI range between 20-30 kg/m^2^ from January 2016, to July 2019, were included consecutively in study. The follow-up period was January 2016, to October 2021. Inclusion criteria; Age between 18–45 years, basic literacy, at least 1-year history of infertility, and female partner age < 35 years, female BMI < 24.5–18 kg/m^2^, with no infertility factor in the female partner of the couple, were included in the study**. **Exclusion criteria; Patients with recent fever, abnormalities of the external genitalia, cryptorchidism, varicoceles, presence of anti-sperm antibodies, taking treatment that can alter spermatogenesis, and patients with chronic diseases e.g., liver/renal disease, patients with hypertension, and diabetes, andrological disorders were excluded from the study. Male BMI below 19.5 and over 30 kg/m^2^. Couples with less than 4 oocytes retrieved or undergoing frozen embryo or frozen sperm treatment were excluded from the study.

### Sample size calculation

The sample size was calculated assumption of z α/2 = equals to 1.96 at confidence level (*α* = 0.05) of 95%, *p* = proportion of population [25–27], and e = margin of error 5% (0.05). The sample size was calculated with a confidence interval of 95% and a precision of 5% (error) using standard formula [28]. *n*= (z-α/2)2 x p x (1-p)/e2 *n*= 1.962*0.28*(1-0.316)/0.052 This sample was inflated by 20% to non-responders (failure to give consent, medical and personal dropouts) and design effect 2, the sample size was: *n*=310 x2 x 20% ~ 750The final total number of couples was 750 with couple male BMI < 24.5–20 kg/m^2^ served as reference group, while male patient with BMI > 24.5-30 kg/m^2^ were considered to be overweight. Overweight male partner were 403, and normal weight were 347, the average age of the female partners was equal to or less than 35 years and BMI less than 24.5 kg/m^2^, while the male partner average age was 38 years old.

### Sampling technique and data collection

The non-probability convenience sample technique was used for couples who underwent IVF/ICSI treatment, with history of primary subfertility were recruited and their data was analyzed. We explained the objective of the study to the participants. We obtained permission for data collection from the fertility clinic. Data collection was also done electronically and following couples’ characteristics were evaluated; age (full years), duration of infertility (years), smoking habits, history of hypertension or diabetes mellitus, family history, obesity, infertility and genetic disease or during the first visit by an informal interview with the couple. The study protocol was developed in accordance with the Helsinki declaration. The study protocol and questionnaire were reviewed and approved by the research committee, department of reproductive physiology Quaid-I- Azam University, Islamabad. The questionnaires were kept anonymous. The data obtained were entered into database by the data collector and trained medical staff. The data collector ensured that the interviews and information collected were confidential. Access to the final data set was restricted to the principal investigator. 1063 couples underwent till oocyte pickup stage, while 750 couples followed and completed ART cycle involving fresh transfer after ovarian stimulation. Reasons for postponed embryo transfer includes; risk of ovarian hyper-stimulation syndrome, elevated progesterone levels (> 1.5 ng/ml), and insufficient endometrium on the trigger day. Data collected during followed up time includes pregnancy rate and live birth rate and were assessed only for fresh embryo transfer to prevent potential confounding bias. Delivery of normal live birth and neonatal weight were analyzed, and cumulative pregnancy outcome among 750 couples with or without achievement of a live birth, outcomes was calculated and analyzed. The study protocol was carried out in accordance to the principles of the declaration of Helsinki [29]. Before data and sample collection couples were assured that their identity will be kept anonymous.

### Body mass index

At first visit weight and height of all couples were measured by the trained nurse. BMI was calculated as weight divided by squared height. All couples were divided into two groups based on male partner BMI according to the classification criteria of the world organization; couples with normal BMI male partner 19–24.5 kg/m^2^ and Overweight male partner > 24.5–30 kg/m^2^.

### Outcomes

Couple with four metaphases (MII) oocyte, their male partner's semen samples were collected and prepared on the same day of oocyte collection and assessed was done on same sample for volume, concentration, motility, morphology, sperm chromatin integrity that was measured by sperm chromatin dispersion assay (SCD), toluidine blue (TB) staining and Chromomycin A3 staining (CMA3). The influence of paternal BMI on embryonic development was assessed, which was classified into; peri fertilization effect (fertilization rate, FR), early/late embryonic development (cleavage rate, CR and blastocyst rate-BR), implantation stage (positive beta hCG), cumulative live birth (CLBR) stage (deliver of at least one live birth- with neonatal birth weight) effect were documented.

### Semen standard parameters analysis

The semen sample was collected by masturbation on the day of oocyte aspiration, after 2–5 days of abstinence, and the collected semen sample was left to liquefy at 37 °C for 30 min before analysis. Each sample was split into two aliquots: one was subjected to analysis for seminal characteristics. Standard semen parameters were assessed according to WHO 2010 standards, to summarize, sperm number was determined using an upgraded Neubauer chamber after proper dilution, and motility was determined using a Leica microscope DM300 scoring at least 100 spermatozoa/slide, and morphology was determined using Diff-Quik stain. Sperm deformity index (SDI) and Teratozoospermic index (TZI) are calculated as described by [30]. Briefly, HOST was performed by mixing 100 µl of semen with 1 ml of the hypoosmotic solution, consisting of sodium citrate and fructose, as indicated by Jeyendran et al. [31]. After a 30 min incubation at 37ºC, 100 spermatozoa were counted under a phase-contrast microscope at 400 × . Only clearly swollen cells of different types were considered positive. For ROS assessment te method was described in detail previously [12]. Briefly, the liquefied semen was centrifuged at 300 g for 7 min, seminal plasma was removed and the pellet of cells was washed in PBS (isotonic solution, pH = 7.4) and spun again and decanted. Washed cells were suspended in PBS to adjust sperm concentration to 1.25 × 106/ml. ROS production was measured after the addition of 10 μl of 5 mM freshly prepared solution of luminol (5-amino-2,3-dihydro-1,4-phthalazinedione, Sigma Chemical Co., St. Louis, MO, USA) in dimethylsulphoxide (DMSO, Sigma Chemical Co.) to 400 μl of spermatozoa suspension. A tube containing 400 μl of PBS and 10 μl of luminol solution served as a blank. Chemiluminescence was measured integrally for 15 min using the Digene DCR-1 single detector luminometer (Digene Diagnostics, Inc., Gaithersburg, MD, USA). Results were expressed in relative light units (RLU) per minute and 20 × 10^6^ spermatozoa.

The other aliquot used for sperm preparation, the final acquired fraction was tested for sperm count and motility and then maintained at 37 °C in the same medium for 15 min’ till used for inseminating oocytes through ICSI or IVF. Sperm DNA fragmentation (SCD), and chromatin maturity (CMA3, TB) were evaluated in selected spermatozoa remaining after oocyte insemination.

### Sperm chromatin dispersion assay (SCD)

The SCD test was measured using a Sperm Nucleus DNA integrity Kit (SCD) (Shenzhen Huakang Biomed Co., Ltd., Shenzhen, China) as reported previously [32] Briefly, the following procedure was performed: 60 μl of semen sample was added to a tube with fluidized agarose and drop was placed on glass-slid and covered with a glass coverslip. The coverslip was removed after 4 min at 4 °C. Acid denaturation for 7 min and lysis for 20 min were carried out successively. Next, the slide was rinsed for 3 min with abundant distilled water and dehydrated in sequential 70%, 90%, and 100% ethanol baths for 2 min each. After Wright's staining, a total of 500 spermatozoa were counted manually to evaluate sperm DNA integrity using bright‐field microscopy per slide. The sperm DNA dispersion was calculated to evaluate the degree of sperm DNA integrity. If the value of SCD is less than 30%, it was considered to be normal.

### Toluidine blue (TB) staining

Chromatin integrity was assessed using the toluidine blue (TB) method [33]. The toluidine blue is a cationic dye. It can bind to the negatively charged phosphate residues of the DNA in the loosely packed chromatin and/or impaired DNA. Two hundred randomly selected spermatozoa per sample were examined under high magnification. The cells were classified into two groups: dark violet cells (TB + cells; abnormal chromatin structure) and light blue cells (TB cells; normal chromatin structure).

### Chromomycin A3 staining

The smears were dried and fixed in Carnoy’s solution at 4 °C for 10 min. The slides were stained in the darkroom with 150 ml of CMA3 (0.25 mg/ ml) in McIlvain buffer for 20 min. After that, the slides were washed in buffer and mounted with buffered glycerol [3, 4]. At least 100 spermatozoa were counted for each sample under the fluorescent microscope with a 460-nm filter, bright yellow-stained chromomycin-reacted spermatozoa (CMA3 +) were considered abnormal, and yellowish green-stained (CMA3 −) were reflected as normal protamination [34]

### Ovarian stimulation, IVF, ICSI, and embryo development

After a long protocol with mid-luteal phase long-acting gonadotropin-releasing hormone analogs (triptorelin, Decapeptly, Ipsen Pharma) followed by an exogenous individual dose of recombinant follicle stimulation hormone r-FSH (Gonal-F, Merk Serono- Germany) were used to induce multiple follicular growths, with starting dose ranging from 150 to 225 IU, according to age, body mass index, antral follicular count, AMH level and response to previous stimulation. The stimulation concentration was titer according to Ovarian response (estradiol level and ultrasound every 2 days, till at least two follicles, reached 17 mm diameter. Finally, At 34–36 h following delivery of human chorionic gonadotrophin u-HCG (IVF-C, LG Lifesciences), oocytes were harvested transvaginally by ultrasound guidance, under general anesthesia sedation, and cultured in human tubal fluid supplemented with 5% human serum albumin (HSA) in a 5% CO2 humidified gas environment at 37 °C. Depending on sperm indices and couples' reproductive histories, oocytes were inseminated using conventional IVF with cumulus oocytes were incubated with 60,000 spermatozoa/oocyte in *in-vitro* fertilization supplemented with HSA -IVF-plus medium (Vitrolife Goteborg-Sweden) or for ICSI, OLYMPUS IX51/71/81/53/73/83 microscope assembled with INTEGRA Ti microinjector was used. Oocytes were assessed at 16–18 h after insemination based on the presence of two pronuclei. Individually fertilized oocytes were sequentially cultivated in G1/G2 plus (Vitrolife Goteborg-Sweden) and incubated in MIRI multiroom incubator (Esco Medical) and scored 40, 62, 88, and 112 h after insemination. The number and shape of nuclei and blastomeres were counted, as well as the percentage and kind of fragmentation [5].

### Data analysis

Data were systematically transferred from medical record and questioner to Microsoft excel 2010. All statistical analyses were carried out using the Statistical Package for Social Sciences (SPSS) 20 IBM (Armonk, NY). Data were expressed as mean ± SD the student’s *t*-tests were selected to conduct the statistics and the Chi-Square test was used to compare the percentage. The men recruited in the present study were divided into 2 groups according to the BMI kg/m^2^. BMI was independent variable, while chromatin maturity parameter, fertilization rate and CLBR were considered dependent variable and value were compared to male BMI. Spearman correlation analysis was performed between the various parameters. For those outcomes that were connected to one or more examined parameters, prediction models were built. Subgroup analyses was performed by the conventional IVF or ICSI. Simple linear regression analysis was conducted to identify the relationship between paternal BMI as independent variable with dependent variable includes; CMA3, successful fertilization and CLBR. We used receiver operating characteristic (ROC) curve analysis to test the accuracy (as the area under the curve, AUC) with a 95% confidence interval, the sensitivity, and specificity as well as to identify cutoff values of the paternal BMI in predicting ART outcomes. Logistic regression was used to estimate adjusted odds ratios (OR) with a 95% confidence interval (CI). To confirm the results and control for potential confounders in the computation of CLBR a cox regression was performed. A *p-*value of < 0.05 was considered to be statistically significant.

## Results

**Fig. 1:**
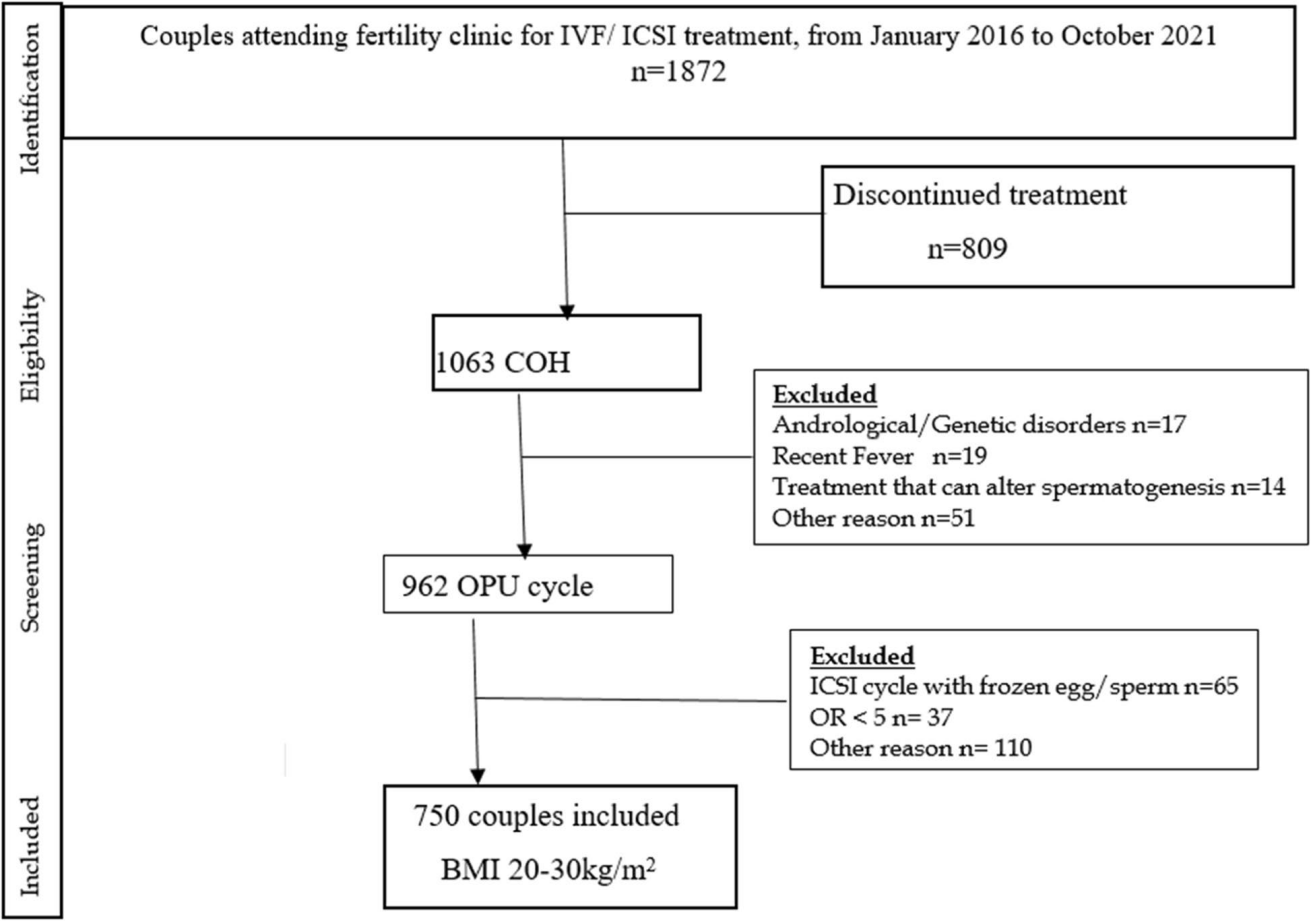
Schematic of the recruitment in the study. COH, Controlled ovarian hyper stimulation; OR, Oocyte retrieve; OPU, Oocyte pick-up; EQ_A_ Embryo with A quality

Fertilization was achieved by conventional IVF (*n* = 290) or ICSI (*n* = 460). A total of 1416 oocytes were fertilized using IVF and 3816 oocytes were fertilized by ICSI. Of all enrolled 1063 couples, infertility information was available underwent their first ovarian stimulations. The percentage of patients under for IVF was normal weight male (24.1%), overweight men (17.6%), while for ICSI was normal weight male (23.2%), Overweight men (34.4%). 750 men there was at least one embryo available for transfer after ART treatment (Fig. 1), thus 21% were considered lost to follow-up. No significant differences were seen in the general characteristics of men who were included in the study as compared to those who were excluded or lost to follow-up.

### Demographic parameters

The mean paternal age of the studied subjects was 38.8 ± 0.25 and paternal BMI was 25.6 ± 0.1 kg/m^2^ (Table 1), the average weight (kg) and height (cm) was significantly higher in over weight men group t (*p* < 0.001). There was no significant difference in demographic parameters analyzed among the normal BMI < 24.5 kg/m^2^ and overweight BMI ≥ 24.5 kg/m^2^ patient groups.

**Table 1:** Age, weight, height, body mass index (BMI), married for, wife age, liquefaction time, volume, PH and WBC in normal weight (BMI < 24.5 kg/m^2^) and overweight (BMI ≥ 24.5 kg/m^2^) and whole studied population

	Normal weight (BMI < 24.5 kg/m^2^) (347)	Over weight (BMI ≥ 24.5 kg/m^2^) (403)	Whole studied population (750)	*p*-Value
Paternal Age (Years)	38.7 ± 0.26	38.9 ± 0.24	38.8 ± 0.25	0.68
Weight (KG)	72.5 ± 0.4	84.07 ± 0.3**	77.8 ± 0.4	< 0.001
Height (cm)	75.49 ± 4.3	95.06 ± 5.07**	86.93 ± 3.31	< 0.001
Paternal BMI(Kg/m^2^)	22.3 ± 0.15	27.6 ± 0.3**	25.6 ± 0.1	< 0.001
Married for (Years)	9.1 ± 0.8	9.4 ± 0.6	9.10 ± 0.7	0.52
Maternal age (Years)	33.1 ± 0.27	33.1 ± 0.29	32.9 ± 0.28	0.33
Maternal BMI(Kg/m^2^) **SEMEN PARAMETERS**				
Liquefaction time (Minutes)	31.75 ± 0.71	31.98 ± 0.46	31.56 ± 0.39	0.06
Volume (ml)	3.85 ± 0.09	3.81 ± 0.1	3.69 ± 0.06	0.39
PH	8 ± 0.0	7.9 ± 0.0	7.97 ± 0.01	0.27
WBC/HPF	4.65 ± 0.32	4.35 ± 0.56	4.52 ± 0.29	0.25

Values represent mean ± SEM

Values in parentheses represent the number of subjects

### Paternal BMI and semen standard parameters

The mean standard conventional semen parameters were comparable between the two groups (Table2). The total normal motile sperms (TNMS) mean was different between group and overweight men had significant (*p* = 0.002) lower number of TNMS (9.32 ± 0.9) compared to normal-weight men (Table 2). The mean levels of ROS were significantly (*p* = 0.001) higher in overweight (BMI ≥ 24.5 kg/m^2^) men than in normal-weight (BMI < 24.5 kg/m^2^) men (Table 2), a ROS level showed a significant positive correlation (*r* = 0.28, *p* < 0.001) with paternal BMI (kg/m^2^) Table 4, while no significant (*p* > 0.05) difference in HOS levels was observed between the two groups.

**Table 2:** Semen parameters analysis in normal weight (BMI > 24.5 kg/m^2^) and overweight (BMI ≤ 24.5 kg/m^2^) men and whole studied population

Sperm Parameters	Normal weight (BMI < 24.5 kg/m^2^) (347)	Over weight (BMI ≥ 24.5 kg/m^2^) (403)	Whole studied population (750)	*p*-Value
Concentration × 10^6^	62.89 ± 3.30	61.47 ± 2.22	62.01 ± 2.23	0.83
Morphology Normal	3.6 ± 0.99	3.5 ± 0.85	3.5 ± 0.65	0.24
TZI	1.81 ± 0.3	1.82 ± 0.3	1.81 ± 0.2	0.67
SDI	1.13 ± 0.3	1.19 ± 0.3	1.17 ± 0.2	0.56
TNMS	11.44 ± 1.5	9.32 ± 0.9	10.75 ± 0.9	0.002
ROS	1.68 ± 0.6	1.9 ± 0.6	1.79 ± 0.4	< 0.001
HOS	53.9 ± 0.9	53.3 ± 0.8	53.7 ± 0.9	0.81
DFI-SCD	22.91 ± 1.23	28.74 ± 1.04	25.38 ± 3.80	< 0.001

Values represent mean ± *SEM*; *TZI* Tetato-zoospermic index, *SDI* Sperm deformity index, *TNMS* Total normal motile sperm, *ROS* Reactive oxygen species, *HOS* Hypo osmatic swelling test-sperm vitality, DFI-SCD, sperm *DNA* fragmentation index – measured by sperm chromatin dispersion assay

Values in parentheses represent the number of subjects

### Sperm chromatin integrity parameters

**Fig. 2:**
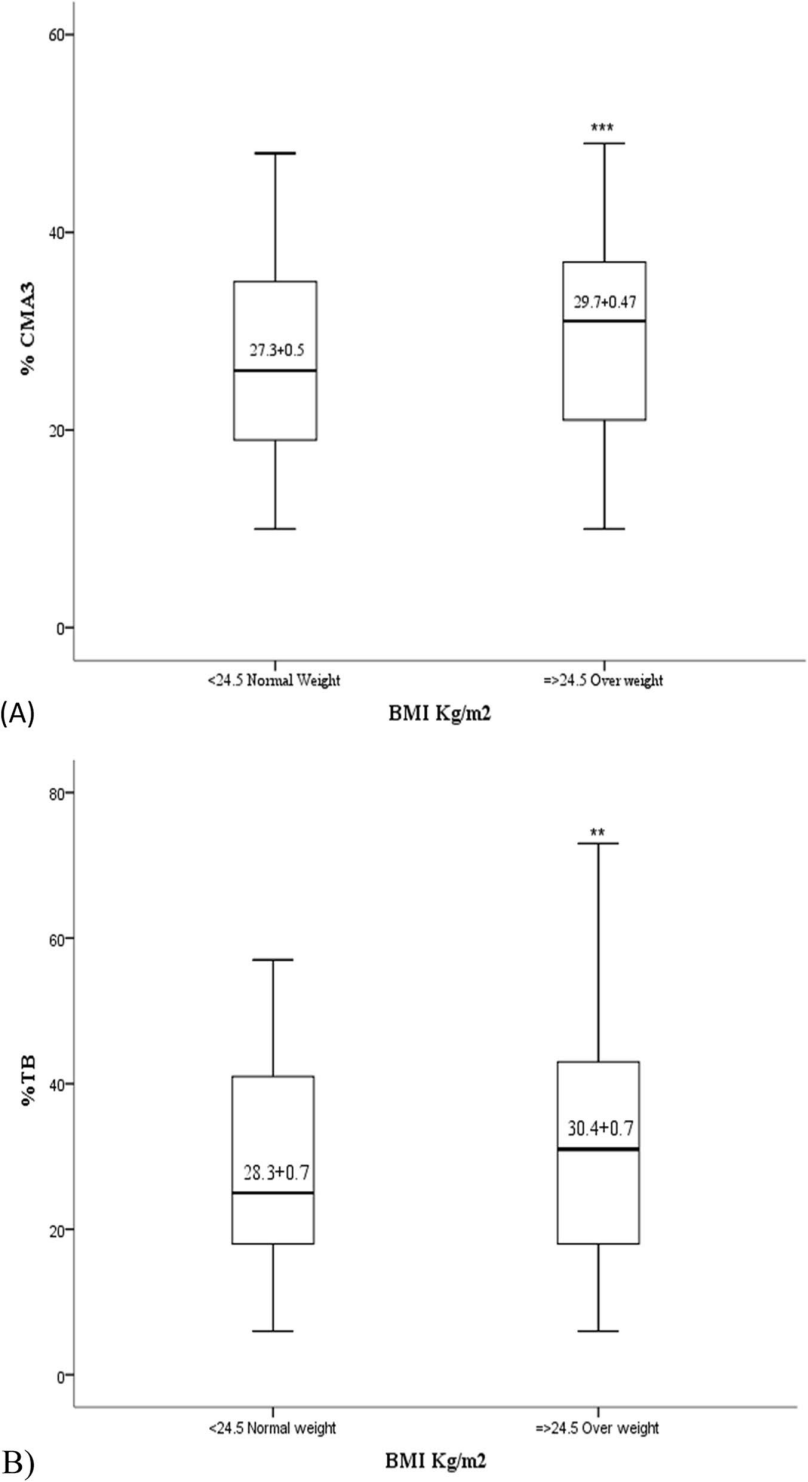
Alteration of sperm molecular parameters in overweight men. A Histones retention as assessed by Chromomycin A3 staining (CMA3) in the sperm of normal weight and overweight men. B Chromatin integrity as assessed by the toluidine blue staining (TB) in the sperm of normal weight and overweight men. (Statistically significant *p < 0.05, **p < 0.01, ***p < 0.001)

**Fig. 3:**
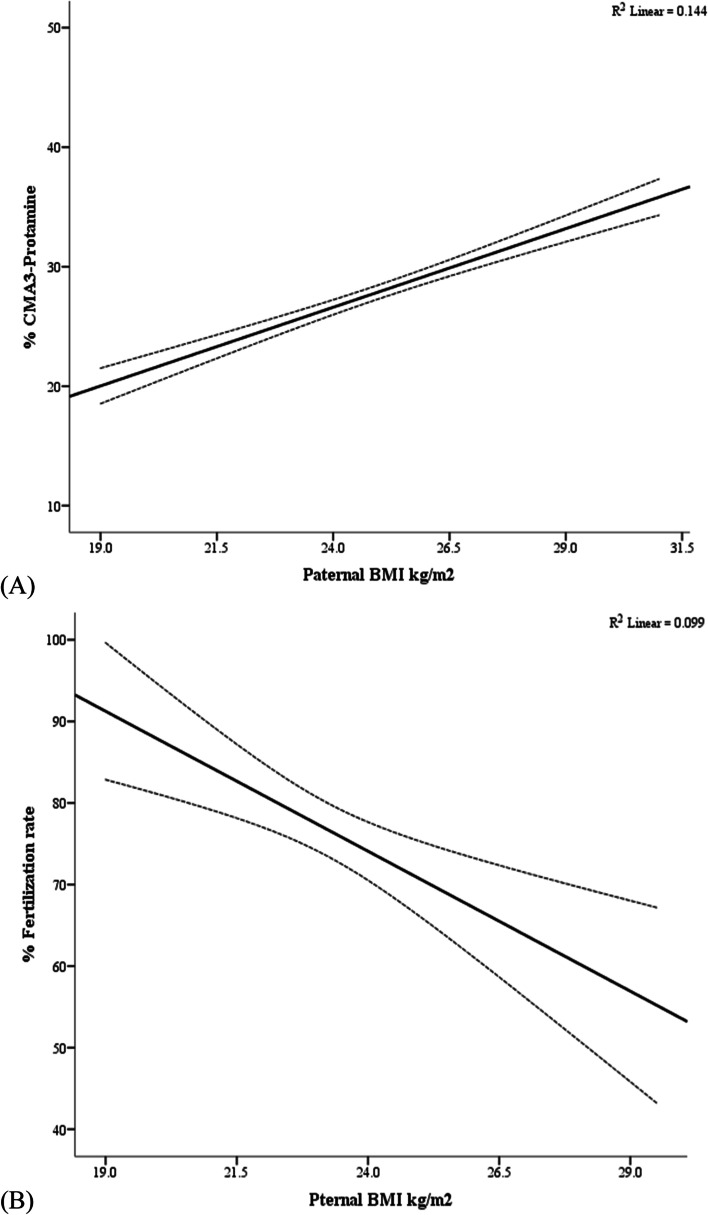
The relationship of (**A**) CMA3, (**B**) Fertilization rate and (**C**) CLBR to body mass. Charts show scatterplots with linear regression lines and their corresponding 95% confidence interval band depicting the association between (A. CMA3, B. Fertilization rate

Overweight men had higher percent of DNA Fragmentation SCD (28.74 ± 1.04) (*p* = 0.001) compared to normal-weight (22.91 ± 1.23) men (Table 2). Mean levels of CMA3 in overweight men were 29.7 ± 9.8 which was significantly (*p* = 0.001) increased than normal-weight men 27.3 ± 9.4 (Fig. 2A), similarly higher (*p* = 0.03) TB levels were observed in overweight men at 30.0 ± 14.4 when compared with normal-weight men 27.7 ± 12.9 (Fig. 2B). Increase in paternal BMI correlate positively with impaired spermatozoa chromatin integrity markers; SCD and TB (*r *= 0.28 and *r* = 0.114, *p* < 0.05) and higher percentage of immature sperm CMA3 (*r* = 0.79, *p* < 0.001) in ejaculate. Univariate linear regression was used to compare paternal BMI and CMA3. After adjustment of female age and number of oocytes MII, a significant positive linear association unmasked between paternal BMI and CMA3 (β = 1.32, CI 95% (1.08 to 1.54); *p* < 0.001) was found with a coefficient of determination (R^2^) equals to 0.144 (Fig. 3A).

### Peri-fertilization stage

The peri-fertilization defects (failed fertilization) were more in the male overweight group, the observed difference was statistically significant (1201/3292, 40% and 687 of 2188 31%, *p* < 0.001) (Table 3). A negative significant correlation was found between paternal BMI and percent fertilization rate (FR) (*r* = -0.187, *p* < 0.001) Table 4. A total 1461 oocytes inseminated by IVF, 1354 (92.7%) oocytes successful to form normal pronuclear (2PN) formation, while 107 (7.3%) oocytes failed to fertilized. Of 3395 oocytes inseminated by ICSI, 546 (16%) oocytes failed to fertilized, while 2849 (83.9%) formed normal 2PN. When cases categorized into BMI categories couple with normal weight had higher fertilization percentage after IVF 878/925 (94.9%) than in overweight men 475/531(89.5% *p* < 0.05). No significant (*p* > 0.05) difference in ART parameters i.e., total egg collected (TEC), metaphase two (MII) and two pronuclei (2PN). A significant decrease in percent fertilization rate (FR) and increase between the overweight (BMI > 24.5 kg/m^2^) men and normal weight (BMI ≤ 24.5 kg/m^2^) men (Table 3). Successful fertilization percentage by ICSI in normal weight men (1218/1514, 80.4%) was significantly (*p* < 0.001) better than in overweight men had (1631/2079, 78.5%,). Univariate linear regression was employed to compare paternal BMI and fertilization rate after ICSI. There was significant inverse linear association between fertilization rate and paternal BMI found after adjustment of female age and number of oocytes MII (β = -1.59, CI 95% (-2.9 to 0.56); *p* = 0.004) with a coefficient of determination (R^2^) equals to 0.099 (Fig. 3B).

**Table 3:** Assisted reproduction parameters and Embryo development outcomes in normal-weight (BMI > 24.5 kg/m^2^) and overweight (BMI ≤ 24.5 kg/m^2^) and whole studied population

Parameters	normal weight (BMI < 24.5 kg/m^2^) (347)	Overweight (BMI ≥ 24.5 kg/m^2^) (403)	Whole studied population (750)	*P*-Value
TEC	8.9 ± 6.4 (2471)	9.2 ± 7.0 (3942)	9.1 ± 6.7 (6413)	0.59
MII	7.7 ± 0.5 (2188)	7.8 ± 0.6 (3292)	7.7 ± 0.6 (5480)	0.83
2PN	5.3 ± 0.7 (1501)	5.2 ± 0.2 (2091)	5.2 ± 0.4 (3592)	0.84
FR	74.8 ± 0.25	67 ± 0.2	70.2 ± 0.8	0.001
D3 embryos	3.8 ± 0.7	4.1 ± 0.4	4.0 ± 0.2	0.31
EG_A_	2.8 ± 0.9	2.7 ± 0.4	2.7 ± 0.6	0.89
CR	82.4 ± 0.24	83.5 ± 0.23	83.1 ± 0.24	0.56
BR	42.5 ± 0.24	39 ± 0.25	40.7 ± 0.24	0.42
Aneuploidy (%)	39.1 ± 2.1	38.1 ± 2.0	38.1 ± 1.4	0.45
IR (%)	131/347 (37%)	143/403 (35%)	274/750 (36%)	0.51
CLBR	31.2 ± 3.4	20.1 ± 3.2	25.2 ± 3.4	0.02
Birth weight (gm)	2577.24 ± 30.94	2952.14 ± 53.64	2752.5 ± 36.98	< 0.001

Values represent mean± *SEM; BMI* Body mass index, *TEC* Total egg collected, *MII* Metaphase two oocyte, *2PN* Two pronuclei, *FR* Fertilization rate, *D3* embryo Day three embryos, *EG*_*A*_, Embryo grade A, *CR* Cleavage rate, *BR* Blastocyst rate, *IR* Implantation rate, *ET* Number of embryo transferred, *CLBR* Cumulative live birth rate

**Table 4:** Correlation between the paternal BMI (kg/m2), CMA3, TB, DFI, HOS, and ROS with ART outcome

		PBMI	CMA3	TB	DFI-SCD	HOS	ROS
Paternal BMI kg/m^2^	r =		0.79^**^	0.114^*^	0.282^**^	0.012	0.282^**^
	*p* =		< 0.001	.030	< 0.001	0.742	< 0.001
FR	r =	-0.187^**^	-0.043	-0.031	-0.062	0.070	-0.148^**^
	*p* =	< 0.001	0.261	0.422	0.107	.068	< 0.001
D3 Embr	r	-0.019	0.075	-0.049	-0.038	0.051	0.021
	*p*	0.70	0.593	0.13	0.45	0.301	0.59
EG_A_	r =	0.052	0.08	0.021	0.030	-0.01	0.06
	*p* =	0.325	0.1	0.693	0.577	0.78	0.259
CR	r =	-0.110^**^	.009	.024	-0.005	-0.023	0.042
	*p* =	0.004	0.814	0.526	0.897	0.556	0.277
BR	r =	0.01	0.07	0.08	0.09	0.23	-0.039
	*p* =	0.96	0.59	0.64	0.49	0.15	0.7
Euploid	r =	0.061	0.060	-0.014	0.007	-0.004	0.045
	*p* =	0.142	0.143	0.740	0.858	0.923	0.279
ET	r =	-0.052	0.011	0.015	-0.003	0.105^**^	0.005
	*p* =	0.157	0.763	0.684	0.939	0.004	0.882
IR	r =	0.42	0.008	0.05	0.027	0.016	-0.062
	*p* =	0.25	0.83	0.17	0.45	0.654	0.09
CLBR	r =	-0.38**	0.002	0.025	-0.09	-0.03	-0.52
	*p* =	< 0.001	0.445	0.97	0.16	0.59	0.44

*BMI*, Body mass index; FR, fertilization rate; D3 embryo (day three embryos), EG_A_, embryo grade A, CR cleavage rate; BR, blastocyst rate, ET, number of embryo transferred; IR, implantation rate; CLBR, Cumulative live birth rate.

Treatment statistically significant *p=0.05, **p=0.01, ***p<0.001

### Early/late embryonic development

Early embryo development includes cleavage rate, day three (D3) embryo, embryo grade A (EG_A_) and late embryo developmental stages of blastocyst formation, euploid embryo and number of embryos transferred we found no significant (*p* > 0.05) difference between two BMI groups (normal weight vs overweight) (Table. 3) and both insemination groups (IVF vs ICSI) (Table 5). We found a significant negative correlation between paternal BMI with cleavage rate (*r* = -0.11, *p* = 0.004), while no correlation was found between paternal BMI and other ART parameters (Table 4).

### Implantation stage

Implantation rate (IR) was 37% in normal weight men group and 35% in over weight men group and there was no difference in both groups Table 3. Sub categories according to insemination (IVF vs ICSI) showed no significant (*p* = 0.25) difference and no correlation was found between male weight and IR (Table 4).

### Outcomes: Cumulative live birth rates

**Fig. 4:**
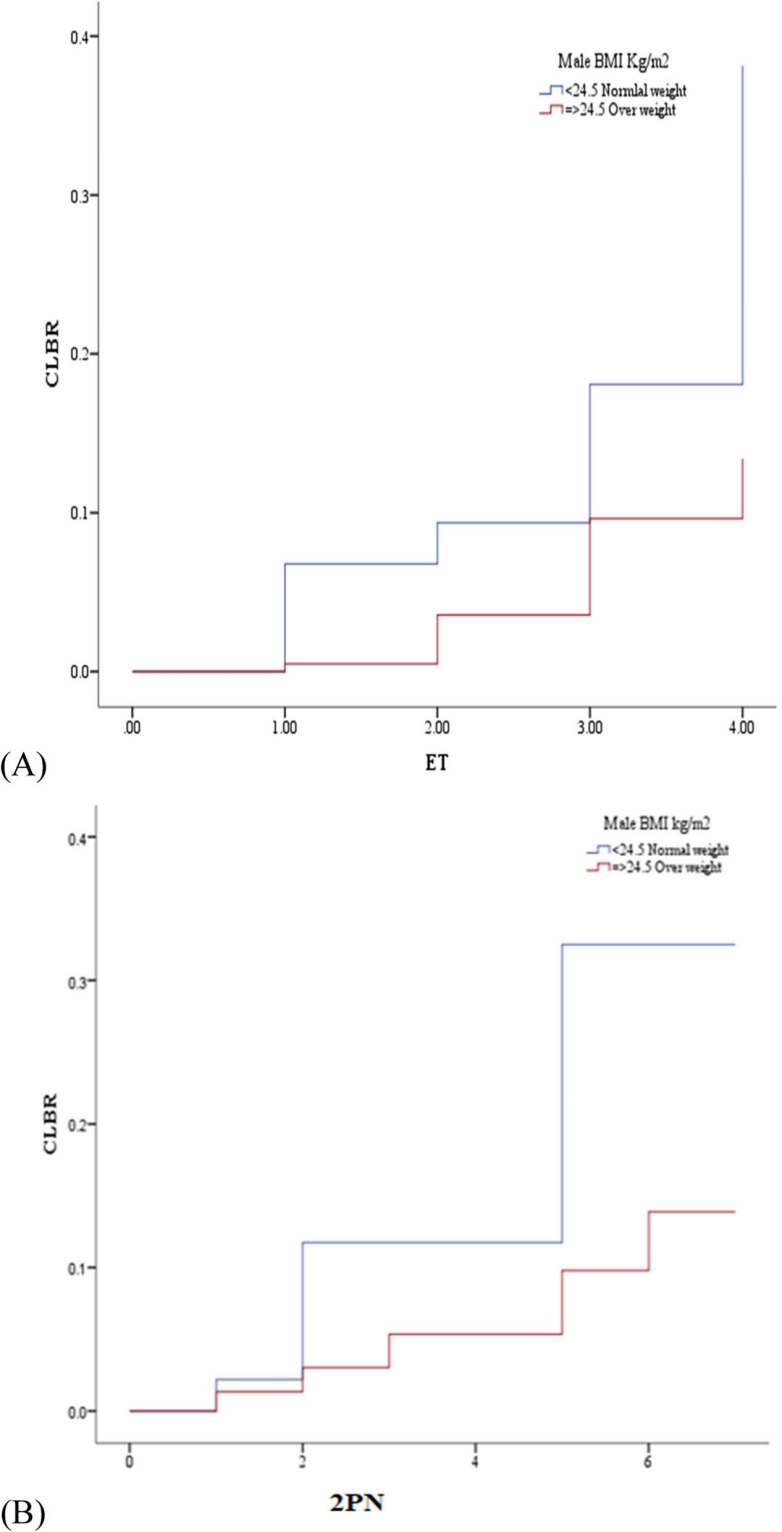
Cumulative live birth rate (CLBR) resulting from the unadjusted analysis of reproductive outcomes in ART cycles using. A CLBR per ET. B CLBR per 2PN (successful fertilization)

In paternal overweight group significant (*p* = 0.02) lower cumulative birth rate (CLBR) compared to reference group Table 3. We found significant increased (with in normal range) neonatal birth weight in paternal overweight group. Sub categories of insemination (IVF vs ICSI) showed no significant difference in CLBR (Table 5). While a significant negative correlation was found between paternal BMI and CLBR (*p* < 0.001). Simple linear regression analysis was conducted, a significant inverse relation identifies between paternal BMI and CLBR (β = -0.67, CI 95% (-0.97 to -0.37); *p* = 0.001) (Fig. 3A). The plotted Kaplan–Meier curves are shown in Fig. 4A and B. The CLBR per embryo transferred in overweight group was 19% for one embryo transferred (ET), 20.6% for two ET, 21.5% for three transfer and for more than three ETs it was 24% while for reference group (normal weight men couples) showed CLBR was 24.6% for one embryo transfer and 25% for both two or three embryo transferred and 24.5% for more than three embryo transferred. The plotted Kaplan–Meier curves are shown in Fig. 4A. The difference between the curves was statistically significant. Cox regression showed a statistically significant negative association between CLBR per ET and paternal over weight (hazard ration (HR) = 5.12, *p* = 0.03) and the CLBR per ET. In case of computing per 2PN (successful fertilization) showed that the reference group (couples with male normal weight) showed CLBR 25.9% for one 2PN, 23.2% for four 2PN and 24.5% for six and more 2PN, while the overweight male couples had CLBR 21.4% for four 2PN, 19.5% for eight 2PN and 24% for more than eight 2PN. The difference between both curves was statistically significant. Consistent with the results of the univariate analysis, the Cox regression showed a statistically significant negative relationship between CLBR per percent fertilization Oocyte (2PN) and paternal weight (HR = 5.3, *p* < 0.05) which was consistent with the result obtained, after adjustment of female age in the univariate analysis (Fig. 4B).

## Discussion

Our study highlighted the impact of men’s overweight on impaired sperm quality and outcome following ART treatment. It is demonstrated that an increase in paternal weight harmed the integrity of sperm chromatin due to elevating reactive oxygen species generation. Our analyses found no influence of paternal BMI on sperm morphology and concentration, while, overweight men had lower motility compared to normal-weight men. Moreover, we found that there have been no statistically significant raises in the spermatozoa deformities index (SDI) in overweight. A statistically significantly higher percentage of normal motile sperm with altered chromatin in overweight men in comparison to normal-weight men was observed, confirming their susceptibility to biological insults, which include weight problems. In this context, a significant negative correlation between paternal BMI and ROS, DFI, CMA3, and TB levels. The paternal weight harmed the integrity of sperm chromatin and its condensation, which represents a higher percentage of immature sperm that could be due to elevated reactive oxygen species generation. These effects are following the findings of previous data suggesting weight gain to be related to higher sperm DNA damage and ROS [10, 14, 17, 18, 35]. Therefore, we can also additionally conclude that being overweight in fathers exerts a negative impact on the molecular components of the motile spermatozoa. An increase in the paternal BMI could lead to impaired sperm chromatin integrity making spermatozoa’s genetic material vulnerable to the external environment insult, as it is understood that sperm chromatin condensation is critical to protect sperm DNA during its transit in the woman's reproductive tract and additionally to manipulate epigenetic reprogramming at some point of the pre-implantation period. Similarly, we observed that an increased in paternal BMI leads to lower fertilization and clinical pregnancy rate after the ART cycle. Hence, Chromatin condensation and DNA integrity are correlated with negative fertility consequences, which might be usually characterized by low fertilization rates, bad embryo quality, repeated failures of assisted reproductive technology attempts, and miscarriages [36–38]. The molecular mechanisms involved in this method are far from being understood at the present, It is thought that after fertilization, the sperm genome undergoes highly-hierarchial epigenetic changes involving the elimination of the sperm nuclear envelope, decondensation of the chromatin via the reduction of the disulfide bonds among protamines, the substitute of paternal protamines by maternal histones, and reprogramming.

The present study we found increased normal neonatal (within normal range) birth weight in the paternal overweight group compared to normal weight. The outcomes of this examination indicated that paternal BMI has an impartial effect on the birth weight of neonates after ART cycles. In ART conception cycles, current information regarding the impact of increased paternal BMI on neonatal birth weight has shown conflicting results [39–41]. Meta-analyses published in 2018 each concluded that ART cycles are constantly related to a decreased risk of a few poor neonatal consequences with low birth weight [39, 40, 42],

Although the underlying mechanisms for the effect of paternal BMI on neonatal outcomes remain unknown, it is quite likely that epigenetic modifications in spermatozoa result in this paternal programming of the neonatal phenotype. Some epigenetic markers in male gametogenesis may also persist at some point in embryonic development. During spermatogenesis, environmental exposures including diet, lifestyle, and different exposures can cause irreversible epigenetic modifications and phenotypic consequences expressed in the following generation. Paternal overweight has been proven to modify the expression of sperm microRNAs and to grow the histone modification and DNA methylation of germ cells. Paternal weight gain can increase histone H3 occupancy in the promoters of the genes involved in embryogenesis and can enhance monomethylation of lysine four on histone H3 (H3K4me1) in genes responsible for embryonic development regulation in spermatozoa. Epigenetic adjustments of the leptin genes in offspring had been discovered in a populace with paternal weight problems earlier than theory. Nonetheless, the consequences obtained from experimental research can't be extrapolated to couples undergoing assisted reproduction [15, 20, 36, 43].

In summary, our examination established paternal overweight is an independent risk component for sperm DNA damage, and chromatin condensation and impacts the reproductive health at pre and post embryological stages of development. The normal weight of female and male partners before in-vitro fertilization is sagacious to increase the quality of gametes, fertilization rate and ART outcome. This finding needs to be confirmed through future large prospective studies.

The present study has some limitations as less number of couples, and not included female factors in the analysis to determine the true influence of the sperm characteristics on ART outcome, as females mask the sperm contribution to embryo development. Moreover, future studies should focus on the association between paternal overweight in infertile couples with idiopathic infertility, because there is clinical concern regarding whether paternal BMI can be used for infertility prognosis.

## Conclusion

The current study demonstrates that the normal weight patients have significantly higher concentration of motile spermatozoa in comparison to overweight patients. However, overweight men had a higher percent of DNA Fragmentation %SCD, significantly higher levels immaturity (Chromomycine staining-CMA3) and lower level of chromatin integrity of spermatozoa as assessed by Toluidine blue (TB) staining. Moreover, mean levels of ROS were significantly higher in overweight men than in normal-weight. In addition, fertilization, cleavage and cumulative birth rate showed a significantly negative correlation with paternal BMI and that paternal overweight is one of the factors contributing to the decline of male fertility. Therefore, cut weight is advisable for patient who undergoing ART therapy.

## Data Availability

All the data are contained in the manuscript.

## Abbreviations

BMI,DNA: Body mass index
DFI: Fragmentation index
MFI: Male factor infertility
SCD: Sperm chromatin dispersion
TB: Toluidine Blue
CMA3: Chromomycin A3
FR: Fertilization rate
CLBR: Cumulative live birth rate
IVF: Invitro-fertilization
ICSI: Intra cytoplasmic injection
ART: Assisted reproduction techniques
ROS: Reactive oxygen species
PGS: Pre-implantation genetic screening
TUNEL: Terminal transferase dUTP nick-end labeling
HCG: Assay, human chorionic gonadotropin
SD: Standard deviation
HOST: Hypo‐osmotic swelling test
SCSA: Sperm chromatin structure assay
TNMS: Total normal motile sperms
TEC: Total egg collected
MII: Metaphase two oocyte
2PN: Two pronuclei
OR: Odds ratios
CI: Confidence interval
COH: Controlled ovarian hyper stimulation
OR: Oocyte retrieve
OPU: Oocyte pick-up
